# The Mayaro virus and its potential epidemiological consequences in Colombia: an exploratory biomathematics analysis

**DOI:** 10.1186/s13071-020-04354-1

**Published:** 2020-10-08

**Authors:** Bryan Steven Valencia-Marín, Irene Duarte Gandica, Oscar Alexander Aguirre-Obando

**Affiliations:** Escuela de Investigaciones en Biomatemática, Universidad del Quindío, Carrera 15, Calle 12 Norte, Armenia, Quindío Colombia

**Keywords:** *Aedes aegypti*, Arbovirus, Biogeographical provinces, *Haemagogus*, Meta-population model (SEI/SEIR), Passive transport

## Abstract

**Background:**

Mayaro virus (*Togaviridae*) is an endemic arbovirus of the Americas with epidemiological similarities with the agents of other more prominent diseases such as dengue (*Flaviviridae*), Zika (*Flaviviridae*), and chikungunya (*Togaviridae*). It is naturally transmitted in a sylvatic/rural cycle by *Haemagogus* spp., but, potentially, it could be incorporated and transmitted in an urban cycle by *Aedes aegypti*, a vector widely disseminated in the Americas.

**Methods:**

The Mayaro arbovirus dynamics was simulated mathematically in the colombian population in the eight biogeographical provinces, bearing in mind the vector’s population movement between provinces through passive transport *via* truck cargo. The parameters involved in the virus epidemiological dynamics, as well as the vital rates of *Ae. aegypti* in each of the biogeographical provinces were obtained from the literature. These data were included in a meta-population model in differential equations, represented by a model structured by age for the dynamic population of *Ae. aegypti* combined with an epidemiological SEI/SEIR-type model. In addition, the model was incorporated with a term of migration to represent the connectivity between the biogeographical provinces.

**Results:**

The vital rates and the development cycle of *Ae. aegypti* varied between provinces, having greater biological potential between 23 °C and 28 °C in provinces of Imerí, biogeographical Chocó, and Magdalena, with respect to the North-Andean Moorland (9.33–21.38 °C). Magdalena and Maracaibo had the highest flow of land cargo. The results of the simulations indicate that Magdalena, Imerí, and biogeographical Chocó would be the most affected regarding the number of cases of people infected by Mayaro virus over time.

**Conclusions:**

The temperature in each of the provinces influences the local population dynamics of *Ae. aegypti* and passive migration *via* transport of land cargo plays an important role on how the Mayaro virus would be disseminated in the human population. Once this arbovirus begins an urban cycle, the most-affected departments would be Antioquia, Santander, Norte de Santander, Cesar (Provinces of Magdalena), and Valle del Cauca, and Chocó (biogeographical province of Chocó), which is why vector control programmes must aim their efforts at these departments and include some type of vector control to the transport of land cargo to avoid a future Mayaro epidemic.
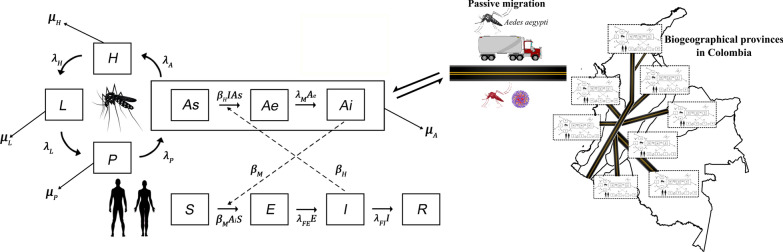

## Background

Mayaro is an arbovirus of the family *Togaviridae* belonging to the genus *Alphavirus*, which includes another 29 viruses; among them, the causal agents of chikungunya fever and the eastern, Venezuelan, and western equine encephalitis virus [1]. Mayaro virus takes its name from the location where it was isolated (reported) for the first time in Trinidad and Tobago in 1954 [2]. Since then, it has been reported in the French Guyana, Brazil, Venezuela, Peru, Bolivia, Surinam, Costa Rica, Guatemala, Panama, Mexico, Haiti, and, sporadically, in Colombia [3, 4]. Since its discovery, three genotypes, D, L and N, have been identified; one of them (L) is exclusive of the Amazon region of the State of Pará, Brazil. The second genotype (D), has been isolated from the Amazon region of Peru, Bolivia, Venezuela, Colombia, Argentina and Brazil, and the third genotype (N) was isolated from a strain from Peru [5, 6].

The disease caused by the Mayaro virus is characterized by high fever and intense joint pain, with duration from 3 to 7 days, causing medical disability [7]. Most infections with Mayaro virus are sporadic and occur in people with a recent history of activity in forest areas (rural or semi-rural) or their surroundings [8]. This disease is not fatal, but it is unknown whether it generates other symptoms, like Zika, which is related with microcephaly in neonates and Guillain-Barré syndrome [9]. The Mayaro virus shares characteristics such as the special tropism through osteomuscular tissue with more prominent arbovirus like dengue (*Flaviviridae*), Zika (*Flaviviridae*) and chikungunya (*Togaviridae*) [10]. In addition, it shares epidemiological, ecological and biogeographic traits, including dependence on vectors at the forest and/or urban level [11–13]. However, unlike dengue (Asian), chikungunya and Zika (African) viruses, it is native to the American continent [8].

The Mayaro virus is endemic in rural areas in South America where it maintains an enzootic sylvan cycle (similar to chikungunya) that involves wild vertebrates (birds and reptiles), including primates and hematophagous mosquitoes [14]. In sylvatic and rural areas, Mayaro is transmitted by the bite of females of the *Haemagogus* spp. infected with this virus [15]. Furthermore, in natural populations of *Aedes* (*Stegomyia*) *aegypti* (Linnaeus, 1762) from South America and North America it has been observed experimentally their susceptibility to being infected and transmitting the genotype D of the Mayaro virus [16, 17]. Additionally, in natural populations of *Ae. aegypti* from South America, natural infection with the genotype L of the Mayaro virus has also been observed [18].

*Aedes aegypti* has the human population as preferential source of food, and urban areas as main reproduction areas [19], although it can also be found in rural areas [20]. Consequently, this species has the potential of serving as vector bridge between rural areas close to natural foci of the Mayaro virus and urban areas [8, 21–23]. Due to the aforementioned, it cannot be discarded that this arbovirus can emerge as a global pathogen through *Ae. aegypti*, as recently observed in chikungunya and Zika arboviruses [24, 25].

The importance of *Ae. aegypti* lies in that it is present in large densities in all continents, with a high degree of anthropophilia, thus, being able to introduce the Mayaro virus into an urban cycle and, thereby, rapidly spread the etiological agent [21, 26]. In the Americas, *Ae. aegypti* is the primary vector of dengue, chikungunya and Zika [27–29], which are distributed in the tropical regions of the continent [21]; in Colombia, this species is present up to 2300 m [30], representing 80% of the territory [9] that could be at risk of contracting the Mayaro virus.

Once the Mayaro virus starts to circulate in the urban area through *Ae. aegypti* and, given that currently no vaccine is available to prevent Mayaro infection in humans (with good results in mice) [31], the only alternative will be the control of this vector. For this purpose, elimination or closing of locations where larvae develop and use of insecticides to control larval and adult forms are ways currently used globally [28]. Nevertheless, frequent use of insecticides has caused resistance to practically all classes of insecticides used globally [32], which is why substitution options by other classes of insecticides are scarce due to the poor offer of environmentally safe products with distinct action sites thus hindering actions to control of this species [33]. Hence, understanding the Mayaro epidemiological cycle in the urban area is of vital importance to elaborate control programmes more in keeping with these new problems.

Given that the Mayaro virus is genetically related to the chikungunya virus [34], and chikungunya is transmitted by *Ae. aegypti* in Colombia, an epidemiological behavior would be expected for Mayaro virus similar to that observed in chikungunya. Recently, it has been observed experimentally that in *Ae. aegypti* individuals infected initially with chikungunya and then with Mayaro virus, both arboviruses can be transmitted with similar efficiency. Further, if it is initially infected with Mayaro virus and then with chikungunya virus, there is an exclusion due to over-infection to chikungunya; in any of the situations described, Mayaro virus will always be transmitted [35].

Since the natural distribution of the Mayaro virus is unknown in sylvatic areas and how it could be potentially transmitted by another vector different from *Haemagogus* spp., an option to know its epidemiological impact is to formulate a mathematical model that permits simulating its behavior and expansion in the human population, through the *Ae. aegypti* potential vector, given that it is distributed in peri-urban and urban areas.

For *Ae. aegypti*, the mathematical models have proven to be useful tools to understand dengue transmission [14, 36] and, recently, chikungunya [37] and Zika [38], as well as to help in planning control strategies [39]. Among the different mathematical approaches to study infectious diseases through vectors, as with the Mayaro virus, the SEIR-type (susceptible, exposed, infected and recovered) epidemiological models [40, 41] and in meta-populations, have been used widely [42–46]. Nonetheless, none of them considers the passive vector transport (dispersion process of invasive species associated with human activities) or its dynamic populations, under different biogeographical conditions; therefore, an option is to include a meta-population model in differential equations, where each local population includes a structured model on age for the dynamic population of *Ae. aegypti* and an SEI/SEIR-type epidemiological model for the population of humans, as well as the passive transport of the mosquito through land cargo, which, has also been used recently to study the dynamics of Rift Valley fever transmission in the human population, with *Ae. aegypti* as the main vector [47]. Among the causes for this heterogeneity, there is the level of connectivity among the places where the species is found and the set of biogeographical conditions where the individuals inhabit [48]. For Colombia, according to Morrone [49], there are eight biogeographical provinces (North-Andean Moorland, Cauca, Napo, Imerí, Chocó, Venezuelan Plains, Maracaibo and western Ecuador), each characterized by having different geographical, climatic, altitudinal and natural components. Among these characteristics, temperature plays an important role in the life-cycle and vector transmission of mosquitoes of medical importance [50, 51]. For instance, optimal temperatures for development, longevity, and fecundity are between 22 °C and 32 °C [52], while at higher temperatures survival range of *Ae. aegypti* decreases; however, egg-laying time increases causing a growth in egg number [53]. Moreover, the extrinsic incubation period of the dengue virus is reduced, resulting in higher rates of viral transmission [54, 55]. On the other hand, at lower temperatures, the transmission of arbovirus may be impeded in some cases [22]. Considering that temperature and passive transport are important factors in arbovirus transmission, here we evaluated how passive transport and the different vital parameters of *Ae. aegypti* affect the dynamics of Mayaro virus transmission in the Colombian human population, from which the study only quantified transport *via* land cargo due to the lack of data from other means of transport.

## Methods

**Fig. 1:**
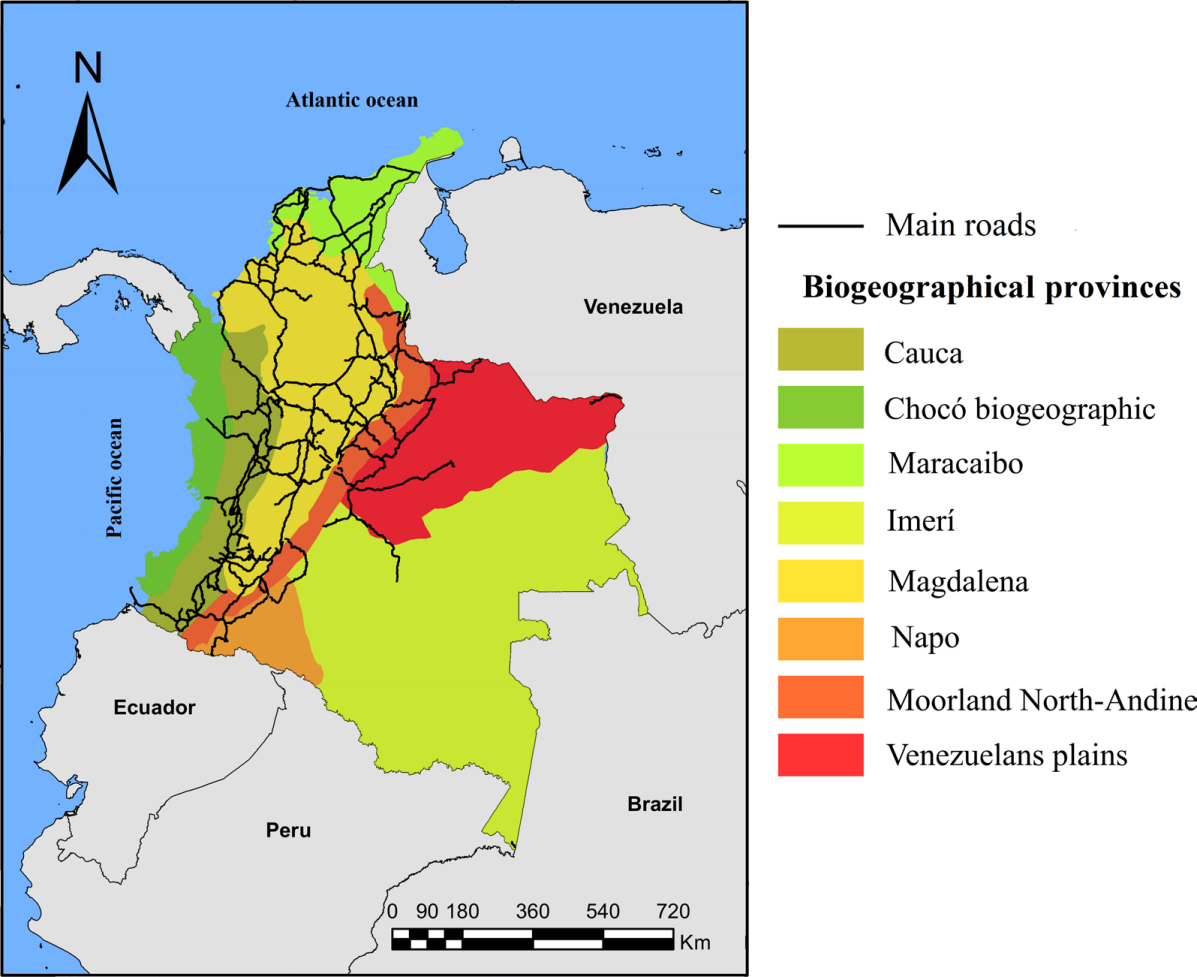
Map of Colombia showing its geopolitical divisions, principal roads, and biogeographical provinces. Biogeographical region Chocó (Pacific coast of northern Ecuador, Colombia and Panama with rainforests and cloud forests), Maracaibo (northern Colombia and northeastern Venezuela with dry forest and rainforest ecosystems and swamps caused by periodic flooding), Magdalena (eastern Venezuela and northeastern Colombia with dry forest and rainforest ecosystems), Venezuelan Plains (Plains covering much of Venezuela and northeastern Colombia, with the largest savanna ecosystem in Latin America), Cauca (western Colombia and Ecuador with rainforests and dry forests), Napo (southeastern Colombia and eastern Ecuador with rainforests, with a vast system of rivers with meanders, which create habitat mosaics), Imerí (southern Venezuela, southeastern Colombia, northeastern Peru, and northern Brazil with diversity of ecosystems, with one of the largest ecosystems of black-water rivers in the world), and the North-Andean Moorland (high mountain ranges (> 3000 m) of Venezuela, Colombia, Ecuador, and Peru, with moorland, pastureland, flood zone vegetation, and cushion vegetation habitats)

The parameters involved in the epidemiological dynamics of the Mayaro virus were obtained from the literature; data used here belong to Mayaro genotype D (Table 1). In a parallel manner, based on the biogeographical regions for Latin America proposed by Morrone [49], Colombia was divided into eight biogeographical provinces (Fig. 1). For each biogeographical province, from historical data available in the Wolrdclim-2 library on temperature from 1970 to 2000 [56], the minimum and maximum median historical temperature was estimated. These variables are recognized in the literature as the most influential in the life-cycle of *Ae. aegypti* [50, 51]. The areas of the biogeographical provinces were defined for Colombia, as proposed by Morrone [49] in R software version 3.5.1, using the *raster*, *rgdal* [57], *sp* [58], *dplyr* [59] and *st* [60] libraries. Thereafter, the layers of the biogeographical provinces were overlapped with each of the raster layers for temperature. Then, for each biogeographical province and each raster layer of temperature, the median, minimum, and maximum historical average temperatures were extracted. At the same time, the vital rates of *Ae. aegypti* corresponding to different temperatures were obtained from the literature and adjusted by average for each region, taking into account its temperature range. Additionally, in 2018 and for each province, the size of the human population was estimated through the projections of the Departamento Administrativo Nacional de Estadística (DANE; Table 1) [61].

**Table 1:** Parameters of the life-cycle of *Ae. aegypti* calculated from the average of the data available in the literature for the temperature range of each biogeographical province and human populations

Biogeographical province	Human population	Temperature (°C)			Transference rate			Survival (days)	Mortality rate			References
		Maximum	Median	Minimum	Egg	Larva	Pupa	Adult	Egg	Larva	Pupa	
Biogeographical region Chocó	1,630,661	30.760	25.740	21.170	0.266	0.121	0.241	28.094	0.088	0.160	0.113	[52, 70, 85, 97, 104–106]
Maracaibo	10,858,909	32.360	26.150	19.910	0.273	0.109	0.247	25.557	0.126	0.135	0.179	[52, 78, 87–89]
Venezuelan Plains	702,579	34.160	26.930	21.000	0.281	0.126	0.277	23.461	0.124	0.135	0.129	[52, 78, 87–89]
Magdalena	9,749,551	30.300	24.430	18.920	0.267	0.105	0.233	26.323	0.116	0.141	0.192	[52, 78, 87–89]
Imerí	369,619	32.250	26.300	20.990	0.273	0.125	0.259	26.776	0.105	0.149	0.109	[52, 78, 87–89]
Cauca	10,021,776	25.610	19.780	14.270	0.221	0.074	0.219	19.639	0.283	0.258	0.352	[52, 78, 87]
Napo	2,880,096	29.870	23.940	18.350	0.273	0.116	0.238	26.397	0.114	0.146	0.144	[52, 78, 87–89]
North-Andean Moorland	13,576,222	21.380	15.300	9.330	0.192	0.063	0.191	17.951	0.375	0.290	0.446	[52, 78, 87]

These data were included in a meta-population model in differential equations, where each province was considered a local population. For each province, a SEI-type model structured on age for the population dynamics of *Ae. aegypti* was combined with a SEIR-type epidemiological model for the human population. In addition, the model included a term that represents the passive transport of the mosquitoes through land cargo in Colombia, using data from the Colombian Ministry of Transportation for 2017 [62].

**Fig. 2:**
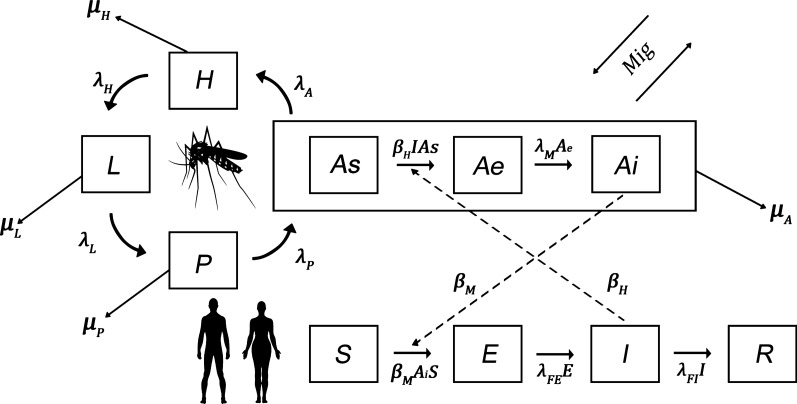
Compartmental diagram of the SEI/SEIR model of the Mayaro transmission through the *Ae. aegypti* vector in the human population. Rectangles indicate the mosquito’s development stages and the Mayaro states of incubation. The arrows around the mosquito image indicate the transitions between each stage. Arrows coming out from each stage indicate the mortality rate in each stage. Broken arrows indicate modulation/influences between the adult mosquito and the disease

Figure 2 represents the compartmental diagram of the model. In the model structured on age for the population dynamics of *Ae. aegypti*, four stages were considered: egg ($H$); larva ($L$); pupa ($P$); and adult (*A*). The adult stage is divided, in turn, into three classes, based on the SEI epidemiological model: susceptible ($As$), exposed ($Ae$), and infected ($Ai$). The vital rates of *Ae. aegypti* in each stage vary according to the biogeographical province (^*r*^). Time $\left( t \right)$ is measured in days.

The variables and parameters used are defined by: $H^{r} \left( t \right)$: number of eggs over time $t$, in province $r$; $L^{r} \left( t \right)$: number of larvae over time $t$, in province $r$; $P^{r} \left( t \right)$: number of pupas over time $t$, in province $r$; $As^{r} \left( t \right)r$: number of adults susceptible over time $t$, in province $r$; $Ae^{r} \left( t \right)$: number of adults exposed over time$t$, in province $r$; $Ai^{r} \left( t \right)$: number of infectious adults over time $t$, in province $r$; $\theta :$ proportion of females/males; $K$: carrying capacity for larvae; $\chi$: intraspecific competition coefficient, proportional to $K$; $\mu_{x}^{r} :$ death rate of individuals of stage $x$ ($x = H,L, P \;and \;A$); $\lambda_{H}^{r}$: rate of transition from egg to larva, in province $r$; $\lambda_{L}^{r}$: rate of transition from larva to pupa, in province $r$; $\lambda_{P}^{r}$: rate of transition from pupa to adult, in province $r$; $\lambda_{A}^{r}$: rate of oviposition of female mosquitoes, in province $r$; $\lambda_{Ae}$: rate of transition from exposed to infected adult mosquitoes; $m_{r}$: net number of adult migrant mosquitoes per day in province $r$; $\beta_{H}$: rate of effective contacts between susceptible mosquitoes and infected humans; $\beta_{M}$: rate of effective contacts between infected mosquitoes and susceptible humans.

The system of differential equations that interprets the dynamics of *Ae. aegypti* is the following:

$$\frac{{dH^{r} }}{dt} = \theta \lambda_{A}^{r} \left( {NM} \right) - \left( {\lambda_{H}^{r} + \mu_{H}^{r} } \right)H^{r}$$

$$\frac{{dL^{r} }}{dt} = \lambda_{H}^{r} H^{r} - \left( {\lambda_{L}^{r} + \mu_{L}^{r} } \right)L^{r} - \chi \left( {L^{r} } \right)^{2}$$

$$\frac{{dP^{r} }}{dt} = \lambda_{L}^{r} L^{r} - \left( {\lambda_{P}^{r} + \mu_{P}^{r} } \right)P^{r}$$

$$\frac{{dAs^{r} }}{dt} = \lambda_{P}^{r} P^{r} - \beta_{H} (I^{r} /NH^{r} )As^{r} - \mu_{A}^{r} As^{r} + m_{r} As^{r}$$

$$\frac{{dAe^{r} }}{dt} = \beta_{H} (I^{r} /NH^{r} )As^{r} - \lambda_{Ae} Ae^{r} - \mu_{A}^{r} Ae^{r} + m_{r} Ae^{r}$$

$$\frac{{dAi^{r} }}{dt} = \lambda_{Ae} Ae^{r} - \mu_{A}^{r} Ai^{r} + m_{r} Ai^{r}$$

$$NM^{r} = As^{r} + Ae^{r} + Ai^{r}$$

The human population was divided into four classes: susceptible ($S$); exposed ($E$); infected ($I$); and recovered ($R$) over time $t$, (in days). The vital rates of the human population were considered the same for all the biogeographical provinces.

The variables and the parameters used in this model are: $$S^{r} \left( t \right)$$: number of humans susceptible over time $$t$$, in province $$r$$; $$E^{r} \left( t \right)$$: number of humans exposed over time $$t$$, in province $$r$$; $$I^{r} \left( t \right)$$: number of humans infected over time $$t$$, in province $$r$$; $$R^{r} \left( t \right)$$: number of humans recovered over time $$t$$, in province $$r$$; $$\lambda_{E}$$: rate of transition from humans exposed to humans infected; $$\lambda_{I}$$: rate of transition from infected to recovered humans; $$\mu_{F}^{r} :$$ death rate of humans in class $$z$$($$F = S,E,R,I)$$; $$N^{r}$$: total human population in province $$r$$ ($$N^{r} = S^{r} + E^{r} + I^{r} + R^{r}$$).

The system in differential equations that interprets the SEIR dynamics for humans is:

$$\frac{{dS^{r} }}{dt} = \mu NH^{r} - \beta_{M} S^{r} \left( {A_{i}^{r} /NM^{r} } \right) - \mu_{F} S^{r}$$

$$\frac{{dE^{r} }}{dt} = \beta_{M} S^{r} \left( {A_{i}^{r} /NM^{r} } \right) - \lambda_{E} E^{r} - \mu_{F} E^{r}$$

$$\frac{{dI^{r} }}{dt} = \lambda_{E} E^{r} - \lambda_{I} I^{r} - \mu_{F} I^{r}$$

$$\frac{{dR^{r} }}{dt} = \lambda_{I} I^{r} - \mu_{F} R^{r}$$

$$NH^{r} = S^{r} + E^{r} + I^{r} + R^{r}$$

The *Ae. egypti* passive transport through the movement of cargo trucks is represented with the term $m_{r} A^{r}$, where $m_{r}$ is the net daily rate of migration of adult mosquitoes between provinces. Assuming that for every 1000 trucks traveling from one province to another, the number of effective migrants $(Mig$) is equal to 3 mosquitoes, as already observed in the field [63]. These numbers are consigned in Table 4, obtained in turn from official data on cargo transport in Colombia from the National Registry of Land Cargo Dispatches (RNDC, for the term in Spanish) (Table 3), and comprise the *Mig* matrix of the 8 × 8 order, whose *ij* component represents the number of individuals going from province *i* to province *j*.

The entry rate of adult individuals in state $X$ (*As*, *Ae*, *Ai*) to province $r$ from other provinces is given by:

$$ImigX\left( r \right) = \mathop \sum \limits_{i = 1}^{8} Mig\left( {i,r} \right)*PropX^{r}$$

The exit rate of adult individuals is state $X$ from province $r$ to other provinces is given by:

$$EmigX\left( r \right) = \mathop \sum \limits_{i = 1}^{8} Mig\left( {r,i} \right)*PropX^{ir}$$

where $PropX^{r} = \frac{{X^{r} }}{{PTA^{r} }}$ is the proportion of adult individuals in state $X$ in province $r$, i.e. the ratio of the number of adult individuals in state $X^{r}$ and the total population of adult mosquitoes $(PTA^{r}$).

The numerical solution of the model was obtained by using the Runge-Kutta 4th order method implemented in MATLAB^®^ environment [64]. From this, and using the parameters obtained from the literature for the life-cycle dynamics of *Ae. aegypti* and the Mayaro virus in each biogeographical province, the behavior of the vector and human populations was simulated in each of the biogeographical provinces, with and without passive transport (migrations), for 300 days. The province of Imerí was taken as center of origin of the infection with Mayaro virus of adult individuals of *Ae*. *aegypti*, given that previous reports have been registered in vertebrates for this arbovirus [65]. Additionally, scenarios were simulated with the beginning of the infection, not in Imerí, but in Magdalena, taking into account that it is the province most connected by land [62]. In both simulations, initial proportions of human populations were taken from DANE data (Table 1), and the initial mosquito populations were hypothetical values because in Colombia and the rest of the world there are no precise estimates of these, there are some mathematical estimates that cannot be extrapolated for our case [66, 67]. In all provinces, the initial populations were *H*: 300, *L*: 200, *P*: 200, *As*: 200 and *Ae*: 0. Relating to *Ai*, an infected mosquito was initially placed in the infection focus region and zero in the others.

A global sensitivity and uncertainty analysis was performed using the partial coefficient correlation index (PRCC). The parameters considered for this analysis were the infection rate from the mosquito to humans ($\beta_{M}$), from humans to the mosquito ($\beta_{H}$) the migration rate ($Mig$), and carrying capacity ($K$) assuming a uniform distribution of the parameters and statistical independence between them. For the uncertainty analysis, the multidimensional parametric space of the parameter values was explored using the Latin hypercube sampling (LHS) [68] method in the *LHS* package [69] using R software. The effect of the variation was analyzed of the parameters on the model response variable, infected adult mosquitoes (*Ai*), through a sensitivity test [70], by means of a partial correlation coefficient analysis (PRCC) implemented with the *sensibility* package [71] in R. Additionally, to determine the population dynamics of *Ai* for those parameters significantly correlated with it, from the data reported, they were simulated taking into account its respective interval scale.

## Results

From the data of the life-cycle dynamics of *Ae. aegypti*, associated with data for temperature from the biogeographical provinces obtained from the literature, it is possible to observe that the provinces with the best conditions for the development of this mosquito species presenting the ranges of most optimal temperatures for the development and life-cycle of *Ae. aegypti*, are Imerí, Chocó and Magdalena (Table 1).

Table 2 shows the parameters involved in the epidemiology of the Mayaro virus, both in the vector such as in humans, obtained also from the literature. These parameters are not related directly with the biogeographical provinces, but with the conditions of the hosts.

**Table 2:** Parameters involved in the Mayaro virus epidemiical dynamics

Parameter	Symbol	Value	References
Duration of the infection in humans		12 (days)	[31, 107]
Intrinsic time of virus incubation		3–11 (days)	[108]
Extrinsic time of virus incubation		7 (days)	[16, 35]
Rate of vector-host effective contact	$\beta_{M}$	0.5–0.8	[109]
Rate of host-vector effective contact	$\beta_{H}$	0.29–0.39	[109]
Larvae carrying capacity	$K$.	50,000	[41, 75]

Data on cargo transport movement available in the RNDC 2017 showed that the provinces with the highest flow of land cargo are Magdalena, Maracaibo and North Andean Moorlands, in contrast with Imerí and Napo which have the lowest connectivity (Table 3). Consequently, the same pattern was observed regarding the daily migration of mosquitoes between biogeographical provinces (Table 4).

**Table 3:** Daily movement of trucks between Colombian biogeographical provinces

Origin/Destination	Chocó	Maracaibo	Magdalena	Venezuelan Plains	Cauca	Napo	Imerí	North-Andean Moorland
Biogeographical region Chocó	0	330.208	1123.085	243.638	1334.058	1014.901	6.745	207.316
Maracaibo	127.984	0	1705.929	42.323	615.268	639.101	2.926	1060.038
Magdalena	20.595	39.430	0	15.310	71.005	258.367	5.573	268.313
Venezuelan Plains	996.636	1415.997	2519.942	0	2216.414	1432.855	47.918	2109.723
Cauca	1217.638	520.868	2264.142	647.249	0	1800.444	42.989	2288.684
Napo	734.860	585.627	1199.586	663.978	1567.970	0	61.460	1041.003
Imerí	1.367	0.507	5.841	1.142	5.532	10.532	0	7.841
North-Andean Moorland	992.189	1244.951	2000.353	852.332	2014.932	1098.164	65.148	0

*Notes*: Data taken from [62]; the vertical list shows the provinces of origin and the horizontal list shows the provinces of destination. Additionally, note that the values along the diagonal are 0 because transport within provinces was not considered

**Table 4:** Passive daily migration of mosquitoes between Colombian biogeographical provinces

Origin	Chocó	Maracaibo	Magdalena	Venezuelan Plains	Cauca	Napo	Imerí	North-Andean Moorland
Biogeographical region Chocó	0	0.991	3.369	0.731	4.002	3.045	0.020	0.622
Maracaibo	0.384	0	5.118	0.127	1.846	1.917	0.009	3.180
Magdalena	0.062	0.118	0	0.046	0.213	0.775	0.017	0.805
Venezuelan Plains	2.990	4.248	7.560	0	6.649	4.299	0.144	6.329
Cauca	3.653	1.563	6.792	1.942	0	5.401	0.129	6.866
Napo	2.205	1.757	3.599	1.992	4.704	0	0.184	3.123
Imerí	0.004	0.002	0.018	0.003	0.017	0.032	0	0.024
North-Andean Moorland	2.977	3.735	6.001	2.557	6.045	3.294	0.195	0

*Notes*: The values presented represent the passive transport of 3 mosquitoes per 1000 trucks, as previously recorded in the literature. Additionally, note that the values along the diagonal are 0 because transport within provinces was not considered

**Fig. 3:**
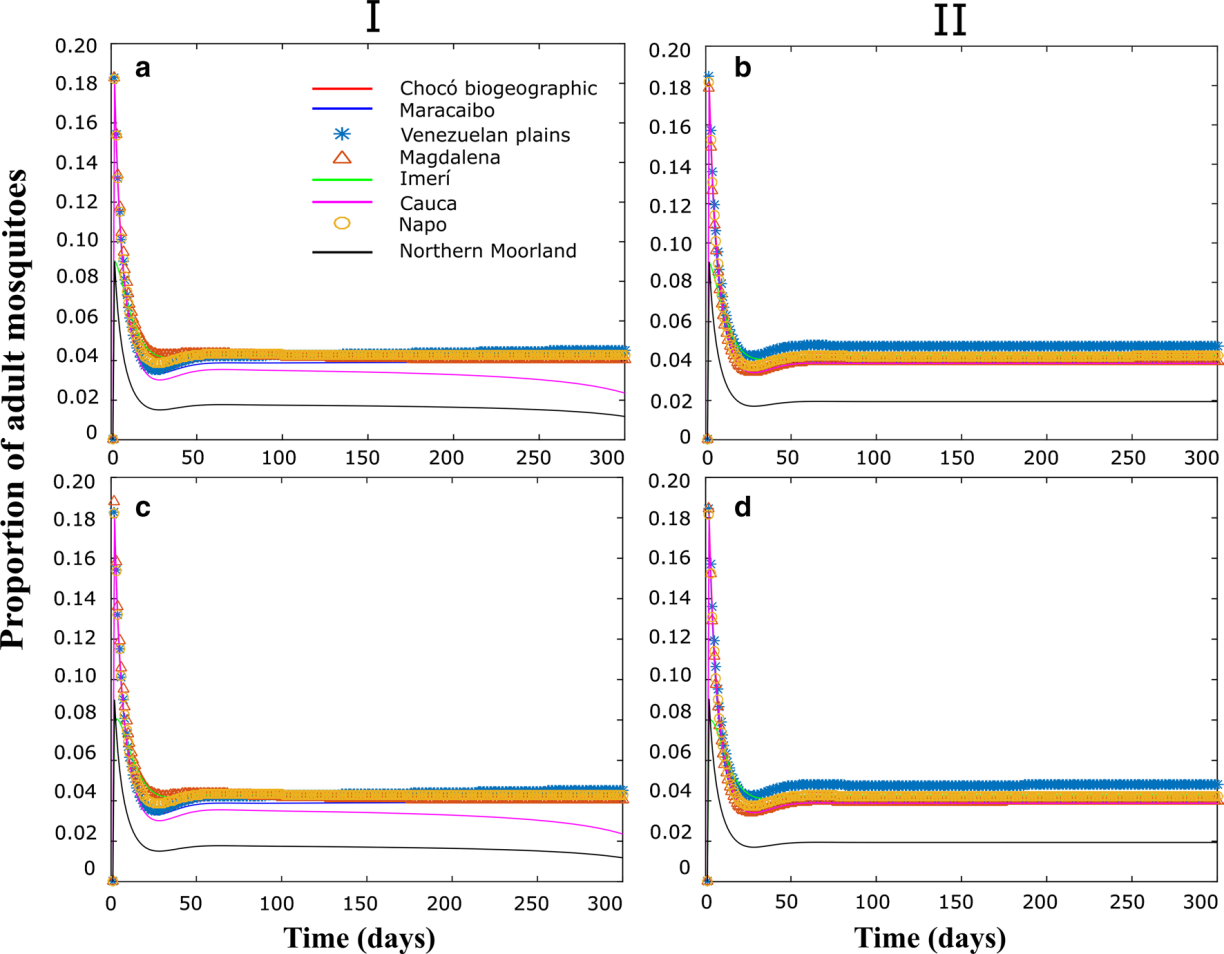
Populations dynamics in proportion of adult *Ae. aegypti* individuals in each biogeographical province, through time. I and II correspond to the simulations with and without migration respectively. **a**, **b** Simulations with the center of the epidemic in Imerí. **c**, **d** Simulations with an epidemic center in Magdalena

**Fig. 4:**
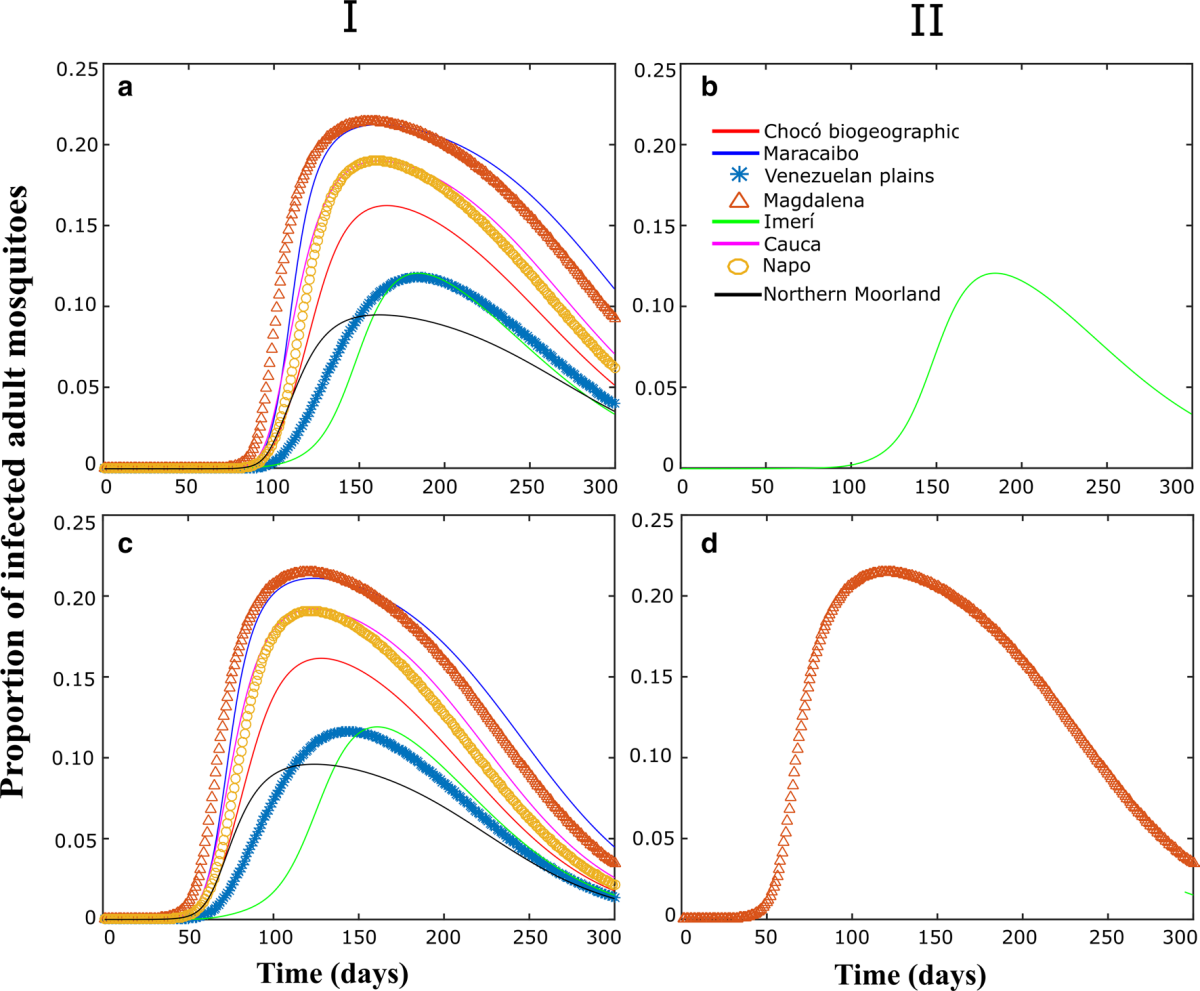
Populations dynamics in proportion of adult mosquitoes infected *Ae. aegypti* individuals in each biogeographical province, through time. I and II correspond to the simulations with and without migration respectively. **a**, **b** Simulations with the center of the epidemic in Imerí. **c**, **d** Simulations with an epidemic center in Magdalena

**Fig. 5:**
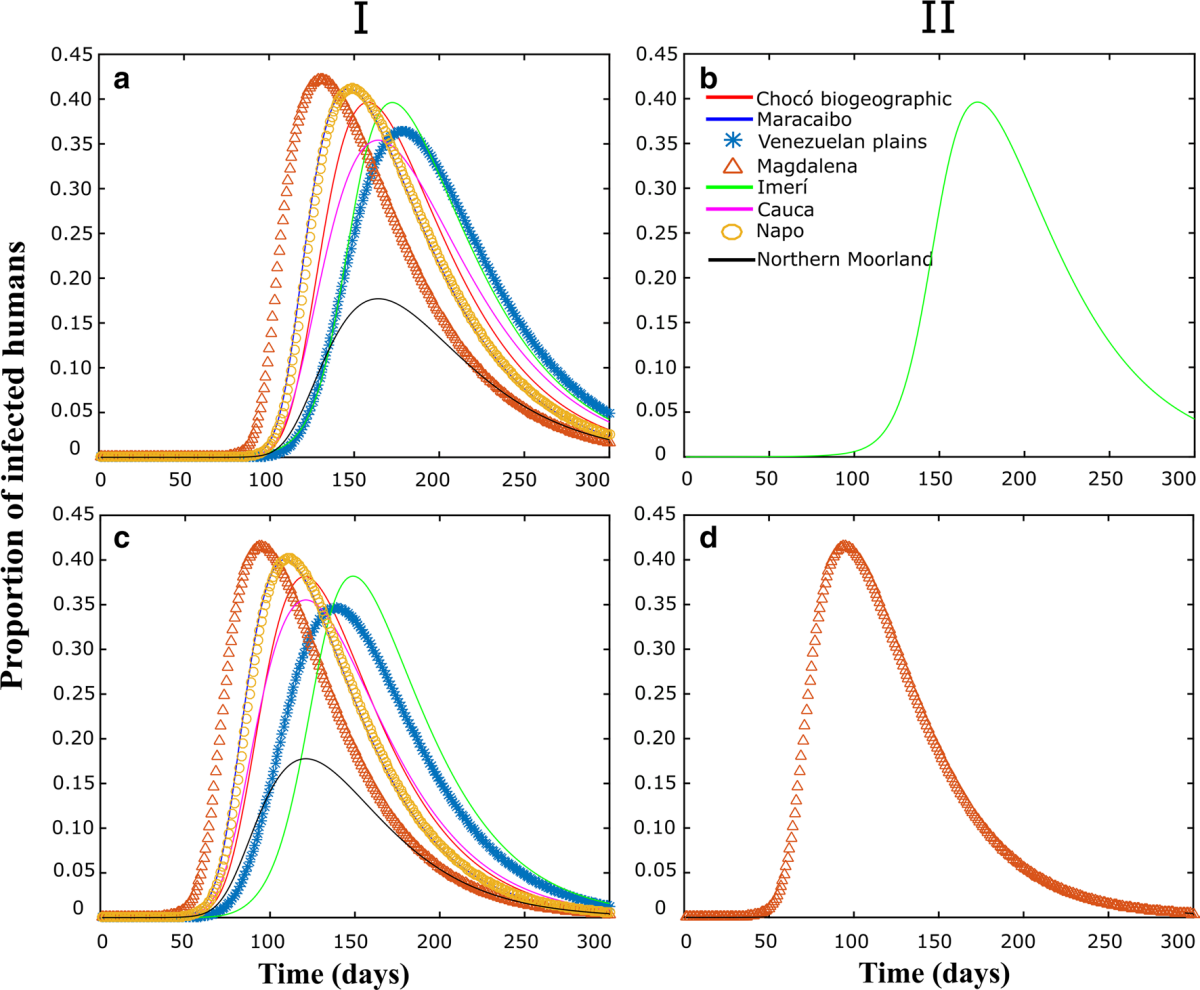
Human infected populations dynamics by Mayaro over time in each biogeographical province under the effect of migration (I) or not (II). **a**, **b** Simulations with the center of the epidemic in Imerí. **c**, **d** Simulations with an epidemic center in Magdalena

Figures 3, 4, 5 correspond to some numerical tests obtained from the model proposed in this study, considering (I) or not (II), passive transport through land cargo and when the focus of infection is in the Imerí region (Figs. 3a, b, 4a, b, 5a, b), which is the province with the lowest connectivity, or in the province of Magdalena (Figs. 3c, d, 4c, d; 5c, d), which is the province with the highest connectivity.

To appreciate the population dynamics corresponding to the different vital rates of each province, Fig. 3 shows the evolution of the proportion of adult mosquitoes in relation to the total population over time in each of the provinces. When passive transport (II) is not considered, the highest values were those of the Venezuelan Plains where the highest transfer rates of immature stages occur, and the lowest values corresponded to the Northern Moorland Province, the province with the highest mortality rate; these dynamics are independent of the infection focus province (Fig. 3b, d). When considering passive transport (I) there were small changes in the dynamics within these regions due to the arrival of individuals from other provinces; similar results were obtained for both scenarios.

To demonstrate the influence of passive transport on the spread of the virus, some simulations are presented here corresponding to scenarios where initially there are only infected mosquitoes in a region; furthermore, the only source of infection considered is due to migrations. Two infection focus regions, Imerí and Magdalena, were chosen, as they had, respectively, the lowest and highest road connectivity with the remaining regions.

Figure 4 corresponds to proportions of infected mosquito populations when the focus of infection is Imerí (Fig. 4a, b) or Magdalena (Fig. 4c, d) and when there is passive transport (I) or it is not considered (II). When migrations are not considered, Fig. 4b and d correspond to the local dynamics in Imerí and Magdalena, respectively; in these cases, the rest of the regions keep the infected population at zero. The most notable difference between the two provinces is the time it takes populations to start growing and the size of the peaks, which is due to the difference between the vital rates of each region. On the other hand, if there is passive transport, infected mosquitoes begin to migrate from the infection focus region, Imerí (Fig. 4a) or Magdalena (Fig. 4c), to neighboring regions and spread according to connectivity between provinces (Table 3) and they are developed following local vital rates (Table 1).

**Fig. 6:**
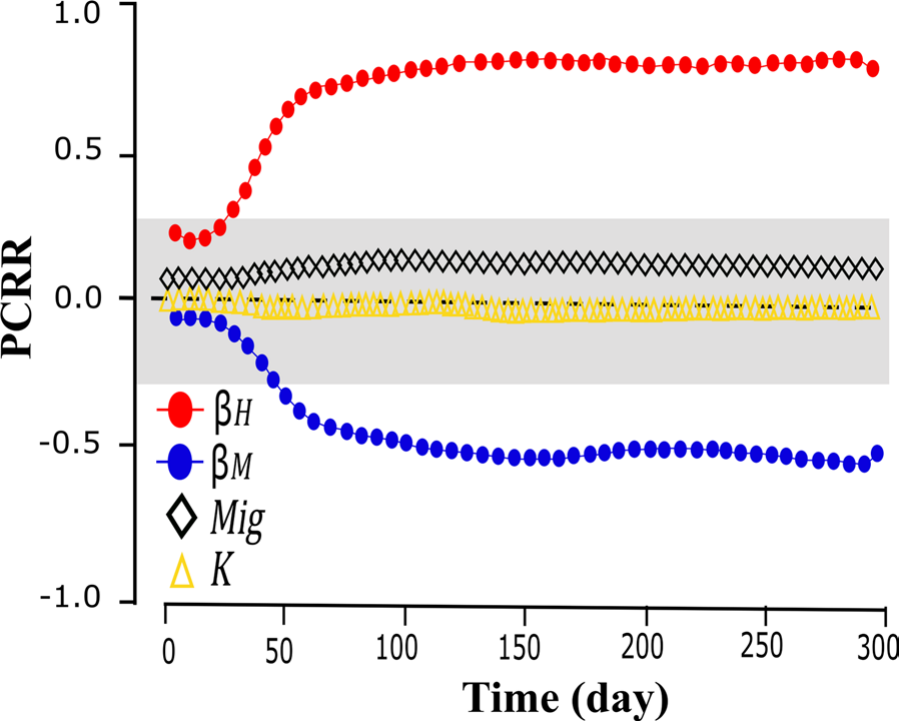
PRCC analysis represents the effect on Imerí of$\beta_{H}$, $\beta_{M}$, $Mig$, and $K$ on the number of mosquitoes infected over time. The gray area represents PRCC values that are not statistically significant

Figure 5 shows the dynamics of infected human populations when the focus of infection is Imerí (Fig. 5a, b) and Magdalena (Fig. 5c, d) and when there is passive transport (I) or not (II). Initially there are no infected humans, so, the dynamics depends on infected mosquitoes. Figure 6 shows the results of the sensitivity analysis made with the parameters human-to-mosquito contagion rate ($\beta_{H}$), mosquito-to-human contagion rate ($\beta_{M}$), number of mosquitoes transported per 1000 trucks ($Mig$) and larvae carrying capacity ($K$). The parameters $\beta_{H}$ (positive) and $\beta_{M}$. (negative) were the only statistically significant and therefore are the parameters that influence this study.

**Fig. 7:**
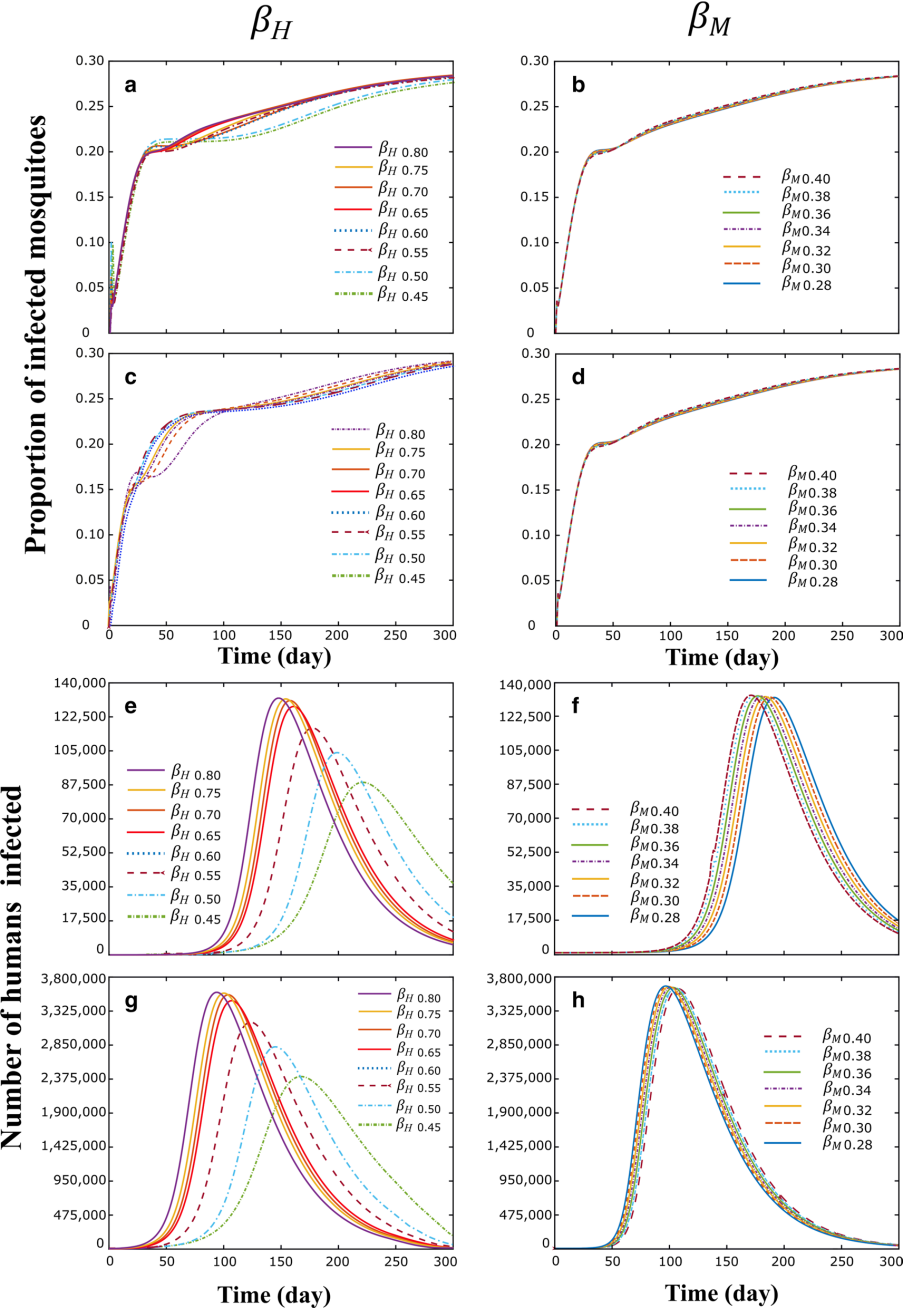
Population dynamic in Imerí and Magdalena providences for the number of infected humans and proportion of infected mosquitoes over time, under the effect of the variation of the $\beta_{M}$ (**a**, **c**, **e**, **h**) and $\beta_{H}$ (**b**, **d**, **f**, **g)** parameters. **a** and **b** correspond to the proportion of mosquitoes infected in the province of Imerí; **c** and **d** correspond to the proportion of mosquitoes infected in the province of Magdalena; **e** and **f** correspond to the number of infected humans in the province of Imerí; **h** and **g** correspond to the number of infected humans in the province of Magdalena

Knowing that $\beta_{H}$ and $\beta_{M}$ are sensitive and significant parameters in the results of the simulations, Fig. 7 shows in the province of Imerí (Fig. 7a, b, e and f) and Magdalena (Fig. 7c, d, g and h), the population dynamic of number of infected humans and the proportion of infected mosquitoes over time, under the effect of the variation of the $\beta_{H}$ (Fig. 7a, c, e and h) and $\beta_{M}$ (Fig. 7b, d, f and g) parameters. In Imerí and Magdalena providences, the population dynamics for infected humans (Fig. 7a, c) and infected mosquitoes (Fig. 7e, h) in the simulations with respect to $\beta_{H}$ parameter showed that with $\beta_{H}$ less than 0.5, both the number of infected humans (Fig. 7a, c) and the proportion of infected mosquitoes (Fig. 7e, h) decrease. In contrast, simulations with different values o. f showed no difference in the behavior of infected humans at Imerí (Fig. 7b) or Magdalena (Fig. 7d) as well as infected mosquitoes at Imerí (Fig. 7f) or Magdalena (Fig. 7g).

## Discussion

Our principal findings indicate that once the Mayaro virus starts to disseminate through *Ae. aegypti* in the vector and human populations, regardless of the center of origin of the infection, Imeri or Magdalena, the provinces of Colombia most affected by the virus will be Magdalena, biogeographical Chocó, and Imerí, having as a basic difference the time in which the infection begins to grow. The epidemiological dynamics of the Mayaro virus observed in the human population is quite similar to that of other arboviruses in Colombia, like dengue between 2004–2013 [72, 73], chikungunya 2014–2015 [74], and Zika 2015–2016 [73], where the most-affected populations were from the departments of Antioquia (6,768,388 inhabitants), Santander (2,100,704 inhabitants), Norte de Santander (1,402,695 inhabitants), Cesar (1,077,770 inhabitants) (Provinces de Magdalena), Valle del Cauca (4,804,489 inhabitants), and Chocó (5,25,296 inhabitants) (Province of biogeographical Chocó).

The dynamics and transmission of this type of arbovirus in human populations, globally, have been associated with the movement through passive human transport and to the colonization of mosquitoes of epidemiological importance such as *Ae. aegypti*, the main vector in the Americas for dengue, chikungunya and Zika [75] and *Ae. albopictus*, the main vector of these arboviruses in Asia and Europe [27, 76]. In *Ae. aegypti*, it has been noted that passive migration occurs principally through human activities, such as movement of land, air, sea and riverine cargo at local, regional and international levels [77, 78], which evidences the urgency to quantify the effect of passive transport on migration [78–80]. Recently, for *Ae. albopictus*, it has been shown that adult individuals are transported passively in private cars and that, for every 1000 vehicles, between two and 11 carry the mosquito [63]. This agrees with the findings in the present study, where the number of individuals for migration to be effective between biogeographical provinces was between one and three mosquitoes for every 1000 trucks. Migration of *Ae. aegypti*, through land cargo transport, influenced directly the migration of this vector and the potential dissemination of the Mayaro virus among biogeographical provinces, principally in Magdalena, with the particularity that when the infection origin center is Magdalena, the Mayaro would spread faster to the other biogeographical provinces compared to when the infection origin center is Imeri. In Colombia, the departments of Antioquia, Cundinamarca, Santander, Norte de Santander and Huila from the province of Magdalena, are the most interconnected and, hence, with the greater probability of migration of mosquitoes infected with the Mayaro virus or not, through cargo transport [62].

Furthermore, changing climate conditions, like global warming, are generating new niches (in latitude, longitude and altitude) and optimal for subsequent habitat expansion not only in *Ae. aegypti* (temperature and precipitation play an important role in the abundance of this species [81]), but also in other species of medical importance [82, 83]. In tropical countries, it has been found that the duration of the development cycle and the vital rates of *Ae*. *aegypti* vary with respect to temperature [84–86], where temperatures between 22–33 °C are optimal for its vital development [52], in contrast to temperatures ≤ 16 °C [87]. Consequently, places with optimal temperatures have population growth and shorter life development for *Ae. aegypti*, as well as increased probability of arbovirus transmission [88], in contrast to places with lower temperatures [87]. Our results show that the provinces of Imerí (20.99–32.25 °C), Chocó (21.17–30.76 °C) and Magdalena (21.17–30.76 °C) have the best conditions for *Ae. aegypti* development, with the highest number of adult mosquitoes at 300 days, in contrast to the province of North-Andean Moorlands (9.33–21.38 °C). Additionally, the carrying capacity (*K*) did affect the population size of living beings; however here, *K* did not affect the population size of *Ae. aegypti*, in these or in the other biogeographical regions, like the size [89] and amount of available water in the larva breeding sites [90], or climatic factors such as humidity and precipitation [91], could be affecting our results; hypotheses that were not tested here. Meanwhile, it should be stressed that these areas in Colombia have the highest levels of precipitation [92]. According to García et al. [93], areas with high levels of precipitation are characterized by higher population densities of *Ae. aegypti* than areas with less rainfall. According to Mohammed & Chadee [94] temperatures ≥ 34 °C cause a gradual drop in the vital rates of *Ae*. *aegypti*, with temperatures > 39 °C being lethal for this species [95, 96], suppressing embryo development and diminishing survival of larval stages [97], having less individuals of the vector in the long term. The only province with temperatures over 34 °C was the Venezuelan Plains, where the vital rates of egg development time, larva, pupa and days of survival of *Ae*. *aegypti* adults are lower with respect to the other provinces.

The model presented here has limitations, which originate from the assumptions considered, among which it was assumed that Mayaro disease is caused by the virus genotype D, the most frequent, and it is dispersed from where there are records of disease outbreaks (Imerí Province [5, 6]), by mosquitoes that are transported by land cargo. However, an additional limitation is the lack of data on Mayaro outbreaks in the other local populations. Despite this, the epidemic dynamics obtained in Colombia is consistent with that reported in other studies available in the literature of dengue [65, 66], chikungunya [67] and Zika [66] for the country.

The HLS/PRCC analysis shows that the contact rate of infected humans and susceptible mosquitoes ($\beta_{H}$) and the contact rate of infected mosquitoes and susceptible humans ($\beta_{M}$) have an effect on the variation in the number of infected mosquitoes over time; this behavior has been previously evidenced by Requena & Juárez [40] in their model for chikungunya suggesting that the most effective way to control or reduce the rate ($\beta_{H}$) is through the use of repellents, mosquito nets, or fumigations at *Ae. aegypti* hatcheries. In this scenario, population control of this vector is essential to prevent a possible Mayaro epidemic; however, currently there are difficulties in controlling *Ae. aegypti* populations due to the spread of resistance to the insecticides used to control it [98, 99]. Therefore, in Colombia, new alternatives for the control of *Ae. aegypti* populations are being evaluated, such as the use and release of mosquitoes infected with *Wolbachia pipientis*-WMel lineage (mosquitoes with refraction to arboviruses transmitted by *Ae. aegypti*) [100]. Additionally, the potential vectorial role of other species such as *Ae. albopictus*, a species that shares ecological niches in Colombia and America with *Ae. aegypti* and that in other latitudes is the main vector of dengue, chikungunya and Zika is unknown [101–103].

## Conclusions

Our results indicate that temperature influences local vector proliferation, since vital rates depend on it. That is, in each biogeographical province the dynamics of the vector is different, while the passive migration of *Ae. aegypti* plays an important role on how the Mayaro virus would be disseminated in human populations. Once this arbovirus begins an urban cycle from the Imerí or Magdalena provinces, the most-affected departments would be Antioquia, Santander, Norte de Santander, Cesar (Provinces of Magdalena), and Valle del Cauca and Chocó (Province of biogeographical Chocó) as already occurred with dengue, chikungunya and Zika. Therefore, vector control programmes must aim their efforts at these departments and include some type of vector control to the transport of land cargo to avoid a future Mayaro epidemic.

## Data Availability

Data supporting the conclusions of this article are included within the article.

## Abbreviations

A: Adult
Ae: Exposed adult mosquito
Ai: Infected adult mosquito
As: Susceptible adult mosquito
E: Egg
L: Larva
P: Pupa
RNDC: National Registry of Land Cargo Dispatches
SEI: Susceptible, exposed and infected
SEIR: Susceptible, exposed, infected and recovered

## References

[1] Serra M (2016). Fiebre por virus Mayaro: una alerta necesaria. Rev Habanera Cien Médi..

[2] Anderson C, Downs W, Wattley G, Ahin N, Reese A (1957). Mayaro virus: a new human disease agent: II. Isolation from blood of patients in Trinidad. Am J Trop Med Hyg..

[3] Lednicky J, Rochars M, Elbadry M, Loeb J, Telisma T, Chavannes S (2016). Mayaro virus in child with acute febrile illness, Haiti, 2015. Emerg Infect Dis..

[4] Zúñiga I, Caro J (2017). Virus Mayaro: una nueva amenaza para el continente Americano. Rev Latin Infectol Pediatr..

[5] Torres J, Russell K, Vasques C, Barrera R, Tesh R, Salas R (2004). Family cluster of Mayaro fever, Venezuela. Emerg Infect Dis..

[6] Auguste A, Liria J, Forrester N, Giambalvo D, Moncada M, Long K (2015). Evolutionary and ecological characterization of Mayaro virus strains isolated during an outbreak, Venezuela, 2010. Emerg Infect Dis..

[7] Tesh R, Watts D, Russell K, Damodara C, Calampa C, Cabezas C (1999). Mayaro virus disease: an emerging mosquito-borne zoonosis in tropical South America. Emerg Infect Dis..

[8] Muñoz M, Navarro J (2012). Virus Mayaro: un arbovirus reemergente en venezuela y latinoamérica. Biomédica..

[9] OPS. MI: Guía de Vigilancia Entomológica y Control del Dengue. 2011. http://new.paho.org/col/index.php?option=com_docman&task=doc_download&gid=1215&Itemid=. Accessed 8 Jul 2018.

[10] Gould E, Pettersson J, Higgs S, Charrel R, Lamballerie X (2017). Emegerging arboviruses: why today?. One Health..

[11] Mejía C, López-Velez R (2018). Alfavirus tropicales artritogénicos. Reumatol Clin..

[12] Gould E, Higgs S (2009). Impact of climate change and other factors on emerging arbovirus diseases. Trans R Soc Trop Med Hyg..

[13] Chen R, Puri V, Fedorova N, Lin D, Hari K, Jain R (2016). Comprehensive genome scale phylogenetic study provides new insights on the global expansion of chikungunya virus. J Virol..

[14] Rodríguez-Morales A, Paniz-Mondolfi A, Villamil-Gómez W, Navarro J (2017). Mayaro, Oropouche and Venezuelan equine encephalitis viruses: following in the footsteps of Zika?. Travel Med Infect Dis..

[15] Llagonne-Barets M, Icard V, Leparc-Goffart I, Prat C, Perpoint T, André P (2016). A case of Mayaro virus infection imported from French Guiana. J Clin Virol..

[16] Long K, Ziegle S, Thangamani S, Hausser N, Kochel T, Stephen H (2011). Experimental transmission of Mayaro virus by *Aedes aegypti*. Am J Trop Med Hyg..

[17] Wiggins K, Eastmond B, Alto W (2018). Transmission potential of Mayaro virus in Florida *Aedes aegypti* and *Aedes albopictus* mosquitoes. Med Vet Entomol..

[18] Serra O, Cardoso B, Ribeiro A, Santos F, Slhessarenko R (2016). Mayaro virus and dengue virus 1 and 4 natural infection in culicids from Cuiabá, state of Mato Grosso, Brazil. Mem Inst Oswaldo Cruz..

[19] Brown J, McBride C, Johnson P, Ritchie S, Paupy C, Bossin H (2011). Worldwide patterns of genetic differentiation imply multiple “domestications” of *Aedes aegypti*, a major vector of human diseases. Proc Biol Sci..

[20] Ngugi H, Mutuku F, Ndenga B, Musunzaji S, Mbayaka J, Aswani P (2017). Characterization and productivity profiles of *Aedes aegypti* (L.) breeding habitats across rural and urban landscapes in western and coastal Kenya. Parasit Vectors..

[21] Kraemer M, Sinka M, Duda K, Mylne A, Shearer F, Brady O (2015). The global compendium of *Aedes aegypti* and *Ae. albopictus* occurrence. Nature..

[22] Halsey S, Siles C, Guevara S (2013). Mayaro virus infection, Amazon Basin Region Peru, 2010–2013. Emerg Infect Dis..

[23] Marrufo M, Sosa N, León J (2017). Fiebre Mayaro: Enfermedad emergente al acecho. Cienc Humanismo Salud..

[24] Powers A, Brault C, Tesh R, Weaver S (2000). Re-emergence of chikungunya and O’nyongnyong viruses: evidence for distinct geographical lineages and distant evolutionary relationships. J Gen Virol..

[25] Volk SM, Chen M, Tsetsarkin KA, Adams AP, Garcia TI, Sall AA (2010). Genome-scale phylogenetic analyses of chikungunya virus reveal independent emergences of recent epidemics and various evolutionary rates. J Virol..

[26] Carvalho F, Moreira L (2017). Why is *Aedes aegypti* Linnaeus so successful as a species?. Neotrop Entomol..

[27] Weaver S (2014). Arrival of chikungunya virus in the new world Prospects for spread and impact on public health. PLoS Negl Trop Dis..

[28] WHO-PES: WHOPES-recommended compounds and formulations for control of mosquito larvae. WHOPES; 2013. WHO Pesticide Evaluation Scheme: “WHOPES”. http://www.hygiene-publique.gov.pf/IMG/pdf/recommandations_oms_larvicides_moustiques_oct_2013.pdf. Accessed 22 Jul 2018.

[29] Yakob L, Walker T (2016). Zikas virus outbreak in the Americas: the need for novel mosquito control methods. Lancet Glob Health..

[30] Ruiz F, Mazo A, Mira A, Gomez G, Zuleta L, Uribe S (2016). Presencia de *Aedes* (*Stegomyia*) *aegypti* (Linnaeus, 1762) y su infección natural con el virus del dengue en alturas no registradas para Colombia. Biomédica..

[31] Choi H, Kudchodkar S, Reuschel E, Asija K, Borole P, Ho M (2019). Protective immunity by an engineered DNA vaccine for Mayaro virus. Plos Negl Trop Dis..

[32] Smith LB, Kasai S, Scott JG (2016). Pyrethroid resistance in *Aedes aegypti* adn *Aedes albopictus*: important mosquito vectors of human diseases. Pestic Biochem Physiol..

[33] Atencia M, Pérez M, Jaramillo M, Caldera S, Cochero M, Bejarono E (2016). Primer reporte de la mutación F1534C asociada con resistencia cruzada DDT y piretroides en *Aedes aegypti* en Colombia. Biomédica..

[34] Fiedrich-jänicke B, Emmerich P, Tappe D, Gunther S, Cadar D, Schanasit S (2014). Genome analysis of Mayaro virus imported Germany from Frech Guiana. Emerg Infect Dis..

[35] Kantor A, Lin J, Wang A, Thompson D, Franz A (2019). Infection pattern of Mayaro virus in *Aedes aegypti* (Diptera: Culicidae) and transmission potential of the virus in mixed infections with chikungunya virus. J Med Entomol..

[36] Gonzalez Morales NL, Núñez-López M, Ramos-Castañeda J, Velasco-Hernández JX (2017). Transmission dynamics of two dengue serotypes with vaccination scenarios. Math Biosci..

[37] Johansson M, Powers A, Pesik N, Cohen N, Staples E (2014). Nowcasting the spread of chikungunya virus in the Americas. PLoS One..

[38] Gao D, Lou Y, He D, Porco T, Kuang Y, Chowell G (2016). Prevention and control of Zika as a mosquito-borne and sexually transmitted disease: a mathematical modeling analysis. Sci Rep..

[39] Winskill P, Harris A, Morgan S, Stevenson J, Raduan N, Alphey L (2014). Genetic control of *Aedes aegypti*: data-driven modelling to assess the effect of releasing different life stages and the potential for long-term suppression. Parasit Vectors..

[40] Requena D, Juárez J (2015). Sugerencias a partir del análisis de sensibilidad de un modelo matemático de transmisión de chikungunya. Rev Peru Med Exp Salud Publica..

[41] Ruiz-Moreno D, Sanchez I, Harrington L (2012). Modeling dinamic introduction of chinkunguya virus in the United States. PLoS Negl Trop Dis..

[42] Lee S, Castillo C (2015). The role of residence times in two-patch dengue transmission dynamics and optimal strategies. J Theor Biol..

[43] Moulay D, Pigné Y (2013). A metapopulation model for chikungunya including populations mobility on a large-scale network. J Theor Biol..

[44] Manrique P, Beir J, Jhonson N (2017). Simple visit behavior unifies complex Zika outbreaks. Heliyon..

[45] Iggidr A, Koiller J, Penna M, Sallet G, Silva M, Souza M (2017). Vector borne diseases on an urban environment: the effects of heterogeneity and human circulation. Ecol Complex..

[46] Mondragón E, Leiton J, Montoya J, Bonilla S (2016). Un modelo multiparche para la transmisión de la malaria. Sigma..

[47] Anzo-Hernández A, Bonilla-Capilla B, Velázquez-Castro J, Soto-Bajo M, Fraguela-Collar A (2019). The risk matrix of vector-borne diseases in metapopulation networks and its relation with local and global R0. Commun Nonlinear Sci..

[48] Gloria-Soria A, Ayala D, Bheecarry A, Calderon-Arguedas O, Chadee D, Chiappero M (2016). Global genetic diversity of *Aedes aegypti*. Mol Ecol..

[49] Morrone J. Biogeografía de América latina y el Caribe. Vol. 3. M&T–Manuales & Tesis SEA; 2001. p. 10–106.

[50] Ewing A, Cobbold A, Purse V, Nunn M, White S (2016). Modelling the effect of temperature on the seasonal population dynamics of temperate mosquitoes. J Theor Biol..

[51] Huber J, Childs M, Caldwell J, Mordecai E (2018). Seasonal temperature variation influences climate suitability for dengue, chikungunya, and Zika transmission. PLoS Negl Trop Dis..

[52] Beserra E, Freitas E, Souza J, Fernandes C, Santos K (2009). Ciclo de vida de *Aedes* (*Stegomyia*) *aegypti* (Diptera, Culicidae) em águas com diferentes características. Iheringia Ser Zool..

[53] Costa E, Santos E, Correia J, Albuquerque J (2010). Impact of small variations in temperature and humidity on the reproductive activity and survival of *Aedes aegypti* (Diptera, Culicidae). Rev Bras Entomol..

[54] Focks D, Brenner R, Hayes J, Daniels E (2000). Transmission thresholds for dengue in terms of *Aedes aegypti* pupae per person with discussion of their utility in source reduction efforts. Am J Trop Med Hyg..

[55] Hopp M, Foley J (2001). Global-scale relationships between climate and the dengue fever vector. Aedes aegypti. Clim Change..

[56] Fick S, Hijmans J (2017). Worldclim 2: New 1-km spatial resolution climate surfaces for global land areas. Int J Climatol..

[57] Bivand R, Keitt Tim, Rowlingson B, Pebesma E, Sumner M, Hijmans R, et al. Package ‘rgdal’. 2015. https://CRAN.R-project.org/package=rgdal.

[58] Pebesma E, Bivand R, Pebesma M, ColorBrewer S, Collate A JTCRAN. Package ‘sp’. 2012.

[59] Wickham H, Francois R, Henry L, Müller K JRFSC, Vienna. https://CRAN.R-project.org/package=dplyr. dplyr: A Grammar of Data Manipulation. R package version 0.4. 3. 2015.

[60] Rainer O, Verena Z, Korbinian S JRFhcrcwpsih. Shrinkage t Statistic and Correlation-Adjusted t-Score, Package “st”. 2015.

[61] DANE: Colombia. proyecciones de población municipales por área 2005. https://www.dane.gov.co/files/investigaciones/poblacion/proyepobla06_20/ProyeccionMunicipios2005_2020.xls. Accessed 19 Dec 2018.

[62] Transporte Md: Registro Nacional de Despacho de Transporte de carga por carretera RNDC. 2017. https://www.mintransporte.gov.co/Publicaciones/atencion_al_ciudadano/servicios_yconsultas_en_linea/registro_nacional_de_despachos_de_carga_por_carretera_rndc. Accessed 18 Oct 2018.

[63] Eritja R, Palmer J, Roiz D, Sanpera-Calbet I, Bartumeus F (2017). Direct evidence of adult *Aedes albopictus* dispersal by car. Sci Rep..

[64] MATHWORKS. MATLAB and Statistics Toolbox Release. The MathWorks, Inc. Massachusetts: USA. 2012.

[65] Pinheiro F, LeDuc J (1998). Mayaro virus disease. Arboviruses Epidemiol Ecol.

[66] Neira M, Lacroix R, Cáceres L, Kaiser P, Young J, Pineda L (2014). Estimación del tamaño de la población de *Aedes aegypti* (Diptera: Culicidae) y la supervivencia de varones adultos en un área urbana de Panamá. Mem Inst Oswaldo Cruz..

[67] Massad E, Akamu M, Bezerra F, Struchiner C, Lopez L, Wilder-Smith A (2017). Estimating the size of *Aedes aegypti* populations from dengue incidence data: implications for the risk of yellow fever outbreaks. Infect Dis Model..

[68] Helton J, Davis F (2003). Latin hypercube sampling and the propagation of uncertainty in analyses of complex systems. Reliab Eng Syst Safe..

[69] Carnell R, Carnell M. Package ‘lhs’. 2019. https://cran.pau.edu.tr/web/packages/lhs/lhs.pdf. Accessed 20 Dec 2019.

[70] Marino S, Hogue I, Ray C, Kirschner D (2008). A methodology for performing global uncertainty and sensitivity analysis in systems biology. J Theor Biol..

[71] Iooss B, Janon A, Pujol G, Iooss M. Package ‘sensitivity’. 2019. http://dk.archive.ubuntu.com/pub/pub/cran/web/packages/sensitivity/sensitivity.pdf. Accessed 20 Dec 2019.

[72] Castrillón J, Castaño J, Urcuqui S (2015). Dengue in Colombia: ten years of database records. Rev Chi Inf..

[73] SIVIGILA: Semana epidemiológica número 52 (25 Diciembre - 31 Diciembre). 2016. https://www.ins.gov.co/buscador-eventos/BoletinEpidemiologico/2016%20Bolet%C3%ADn%20epidemiol%C3%B3gico%20semana%2052%20-.pdf. Accessed 5 Aug 2019.

[74] SIVIGILA: Semana epidemiológica número 52 (27 dic. - 02 ene.). 2015. https://www.ins.gov.co/buscador-eventos/BoletinEpidemiologico/2015%20Boletin%20epidemiologico%20Semana%2052.pdf. Accessed 2 Aug 2019.

[75] Carvajal J, Honorio N, Díaz S, Ruiz E, Asprilla J, Ardila S (2016). Detección de *Aedes albopictus (*Skuse) (Diptera: *Culicidae*) en el municipio de Istmina, Chocó, Colombia. Biomédica..

[76] Rúa-Uribe G, Suárez-Acosta C, Rojo R (2012). Implicaciones epidemiológicas de *Aedes albopictus* (Skuse) en Colombia. Rev Fac Nac Salud Pública..

[77] Goncalves da Silva A, Cunha I, Santos W, Luz S, Ribolla P, Abad-Franch F (2012). Gene flow networks among American *Aedes aegypti* populations. Evol Appl..

[78] Diaz-Nieto L, Chiappero M, de Astarloa C, Maciá A, Gardenal C, Berón C (2016). Genetic evidence of expansion by passive transport of *Aedes* (*Stegomyia*) *aegypti* in eastern Argentina. PLoS Negl Trop Dis..

[79] Huber K, Loan L, Chantha N, Failloux A (2004). Human transportation influences *Aedes aegypti* gene flow in Southeast Asia. Acta Trop..

[80] Gorrochotegui-Escalante N, Gomez-Machorro C, Lozano-Fuentes S, Fernandez-Salas I, Munoz M, Farfan-Ale JA (2002). Breeding structure of *Aedes aegypt*i populations in Mexico varies by region. Am J Trop Med Hyg..

[81] Wang L, Xiang L (2014). Spatial epidemiology of networked metapopulation: an overview. Chin Sci Bull..

[82] Patz J, Daszak P, Tabor G, Aguirre A, Pearl M, Epsein J (2004). Unhealthy landscapes: policy recommendations on land use change and infectious disease emergence. Environ Health Perspect..

[83] Ochoa M, Castellanos R, Ochoa Z, Oliveros M (2015). Variabilidad y cambio climáticos: su repercusión en la salud. Medisan..

[84] Rueda L, Patel R, Axtell R, Stinner R (1990). Temperature-dependent development and survival rates of *Culex quinquefasciatus* and *Aedes aegypti* (Diptera: Culicidae). J Med Entomol..

[85] Domínguez M, Ludueña Almeida FF, Almirón W (2001). Dinámica poblacional de *Aedes aegypti* (Diptera: Culicidae) en Córdoba capital. Rev Soc Entomol Argent..

[86] Campbell L, Luther D, Moo-Llanes J, Ramsey R, Danis L, Peterson A (2015). Climate change influences on global distributions of dengue and chikungunya virus vectors. Philos Trans R Soc Lond B Biol Sci..

[87] Carrington B, Seifert N, Willits N, Lambrechts L, Scott T (2013). Large diurnal temperature fluctuations negatively influence *Aedes aegypti* (Diptera: Culicidae) life-history traits. J Med Entomol..

[88] Muturi E, Blackshear M, Montgomery A (2012). Temperature and density-dependent effects of larval environment on *Aedes aegypti* competence for an alphavirus. J Vector Ecol..

[89] Lega J, Brown E, Barrera R (2017). *Aedes aegypti* (Diptera: Culicidae) abundance model improved with relative humidity and precipitation-driven egg hatching. J Med Entomol..

[90] Valdez L, Sibona G, Condat C (2018). Impact of rainfall on *Aedes aegypti* populations. Ecol Model..

[91] Anna S, Sadie R, Beltrán M, Mercy Á (2013). Dengue vector dynamics (*Aedes aegypti*) influenced by climate and social factors in Ecuador: implications for targeted control. PLoS ONE..

[92] Peraza W. Distribución de la temperatura media anual (°C). Promedio multianual 1981–2010 (IDEAM). 2014. http://atlas.ideam.gov.co/visorAtlasClimatologico.html. Accessed 12 June 2019.

[93] García C, García L, Espinosa-Carreón L, Ley C (2011). Abundancia y distribución de *Aedes aegypti* (Diptera: Culicidae) y dispersión del dengue en Guasave Sinaloa, México. Rev Biol Trop..

[94] Mohammed A, Chadee D (2011). Effects of diurnal temperature regimens on the development of *Aedes aegypti* (L.) (Diptera: Culicidae) mosquitoes. Acta Trop.

[95] Mourya D, Yadav P, Mishra A (2004). Effect of temperature stress on immature stages and susceptibility of *Aedes aegypti* mosquitoes to chikungunya virus. Am J Trop Med Hyg..

[96] Couret J, Dotson E, Benedict M (2014). Temperature, larval diet, and density effects on development rate and survival of *Aedes aegypti* (Diptera: Culicidae). PLoS ONE..

[97] Marinho R, Beserra E, Bezerra-Gumao A, Porto V, Olinda R, Santos C (2015). Effects of temperature on the life cycle, expansion, and dispersion of *Aedes agypti* (Diptera: Culicidae) in three cities in Paraiba, Brazil. J Vector Ecol..

[98] Boëlle P, Thomas G, Vergu E, Renault P, Valleron A, Flahault A (2008). Investigating transmission in a two-wave epidemic of chikungunya fever, Reunion Island. Vector Borne Zoonotic Dis..

[99] Parola P, De Lamballerie X, Jourdan J, Rovery C, Vaillant V, Minodier P (2006). Novel chikungunya virus variant in travelers returning from Indian Ocean islands. J Emerg Infect Dis..

[100] Aliota M, Walker E, Yepes A, Velez I, Christensen B, Osorio J (2016). The wMel strain of *Wolbachia* reduces transmission of chikungunya virus in *Aedes aegypti*. PLoS Neglect Trop..

[101] Johnson D, Druce J, Chapman S, Swaminathan A, Wolf J, Richards J (2008). Chikungunya virus infection in travellers to Australia. Med J Aust..

[102] Dubrulle M, Mousson L, Moutailler S, Vazeille M, Failloux A (2009). Chikungunya virus and *Aedes* mosquitoes: saliva is infectious as soon as two days after oral infection. PLoS ONE..

[103] Vazeille M, Martin E, Mousson L, Failloux AB (2011). Chikungunya, a new threat propagated by the cosmopolite *Aedes albopictus*. BMC Proc..

[104] Yusoff N, Budin H, Salemah I (2012). Simulation of population dinamics of *Aedes aegypti* using climate dependent model. World Acad Sci..

[105] Castro F, Martins F, Lucena L, Almeida R, Beserra E (2013). Ciclos de vida comparados de *Aedes aegypti* (Diptera, Culicidae) do semiárido da Paraíba. Iheringia Ser Zool..

[106] Elia Quispe, Aida Carbajal, Janeth Gozzer, Bertha Moreno (2015). Ciclo biológico y Tabla de Vida de *Aedes aegypti*, en laboratorio: Trujillo (Perú), 2014. REBIOLEST..

[107] Suhrbier A, Jaffar-Bandjee M, Gasque P (2012). Arthritogenic alphaviruses - an overview. Nat Rev Rheumatol..

[108] Esposito D, Fonseca B (2017). Will Mayaro virus be responsible for the next outbreak of an arthropod-borne virus in Brazil?. Braz J Infect Dis..

[109] Dumont Y, Chiroleu F, Domerg C (2008). On a temporal model for the Chikungunya disease: modeling, theory and numerics. Math Biosci..

